# Scaling-up tuberculosis preventive therapy to HIV-negative contacts of bacteriologically confirmed TB cases aged 5 years and above – Lessons from a tuberculosis preventive therapy surge activity in Zambia

**DOI:** 10.1371/journal.pgph.0005628

**Published:** 2026-09-18

**Authors:** Phallon Blessing Mwaba, Innocent Mwaba, Minyoi Mubita Maimbolwa, Chitalu Chanda, Muhammad Mputu, Chola Lumbwe, Kevin Zimba, Nancy Kasese Chanda, Rhehab Chimzizi, Angel Mubanga, Monde Muyoyeta, Mary Kagujje

**Affiliations:** 1 Department of Tuberculosis and other Respiratory Diseases, Centre for Infectious Disease Research in Zambia, Lusaka, Zambia; 2 Department of Public Health, National Tuberculosis and Leprosy Programme, Ministry of Health, Lusaka, Zambia; 3 Department of Health Management and Health Economics, University of Oslo, Oslo, Norway; 4 The Health Coordination Office, United States Embassy, Lusaka, Zambia; Federal University of Rio de Janeiro, BRAZIL

## Abstract

While tuberculosis preventive therapy (TPT) coverage among people living with HIV in Zambia has improved, uptake among HIV-negative contacts aged ≥5 years remains low. Despite evidence showing that TPT can reduce TB incidence by up to 60% in HIV-negative individuals, there is a paucity of evidence on programmatic rollout to this population in Zambia and the region. We present programme data from a TPT surge aimed at increasing uptake among ≥5 years old HIV-negative household TB contacts. The National TB Programme, with support from the TB Local Organizations Network, conducted a TPT surge in 89 high TB-burden facilities across eight project supported provinces in Zambia from October 2024–15^th^ January 2025. After a thorough planning phase, multidisciplinary teams conducted field visits for contact tracing and initiated TPT for eligible household contacts of bacteriologically confirmed TB.. Facility-based TPT initiation was provided for walk-in clients. We collected and analyzed aggregate data from facility registers. A total of 19,371 HIV-negative contacts aged ≥ 5 years were screened: 99.9% (n = 19,344) were eligible for and offered TPT. Of these, 72.4% (n = 14,000) were initiated on TPT, 2.0% (n = 385) refused and 26.2% (n = 4,959) were not initiated on treatment due to unavailability of their preferred regimen. The start of the surge was associated with an immediate 47.7% points increase in TPT coverage (p = 0.001). Overall, 63.7% (n = 8,917) were initiated on three-months Isoniazid+Rifapentine, followed by six-months Isoniazid (30.2%, n = 4226); one-month Isoniazid+Rifapentine (4.5%, n = 625) and on three-months Isoniazid+Rifampicin (1.7%, n = 232). The surge demonstrated that TPT can be rolled out to ≥5 years old HIV-negative TB contacts with high initiation rates achieved. This is achievable through targeted implementation, stakeholder engagement, and community-driven approaches. These findings underscore the feasibility of extending TPT to all TB contacts, regardless of HIV status or age, to accelerate TB prevention efforts in high-burden settings like Zambia.

## Introduction

Zambia is among the top 30 high TB burden countries globally, with a tuberculosis (TB) incidence of 283/100,000 population [1]. The country has made remarkable progress in lowering the TB burden in people living with HIV (PLHIV) with the TB incidence reducing from 205 per 100,000 in 2018–91 per 100,000 in 2023 in this population [2]. However, there has been a shift of the TB burden to the HIV negative population. Nearly 70% of the total TB notifications in Zambia in 2023 were HIV negative [1].

In 2018, Zambia’s Ministry of Health (MoH) adopted the World Health Organisation (WHO) recommendation to extend TB preventive Therapy (TPT) delivery beyond PLHIV and under 5 contacts of TB patients to include HIV negative contacts, aged 5 years and above, of bacteriologically confirmed TB patients and other high risk groups for TB [3]. Similar to other settings, and in spite of strong evidence that TPT can reduce TB incidence by up to 60% in HIV-negative individuals [4–6], its implementation has been slow and variable [7]. Between 2018 and 2021, Zambia made significant progress in TPT implementation, achieving a 90% TPT uptake among PLHIV [8]. However, the coverage among HIV negative household contacts to bacteriologically confirmed TB patients remains much lower, at 55% [1], and these mostly comprise children below 5 years of age.

To address the current gaps in TPT implementation, the national TB program developed the implementation roadmap for TPT, aimed at saturating and sustaining TPT in PLHIV, accelerating TPT uptake in under 5 contacts to TPT cases and increasing focus on other high-risk categories such as all contacts to bacteriologically confirmed TB patients regardless of age and HIV status [9]. In alignment with the roadmap, The United States Government funded Tuberculosis Local Organisations Network (TBLON) project implemented a TPT Surge aimed at scaling up TPT to HIV negative contacts to bacteriologically confirmed TB patients aged ≥5 years. We present programme data from this surge to demonstrate the reach in improving TPT initiation coverage, feasibility and acceptability of TPT among HIV-negative household contacts aged ≥5 years, to inform countrywide scale-up.

## Methodology

### Intervention design and setting

We implemented a TPT surge in routine settings between 1^st^ October 2024 and 15^th^ January 2025 in 89 high TB burden TBLON supported public health facilities in eight out of Zambia’s ten provinces (Southern, Lusaka, Central, Muchinga, Copperbelt, Northern, Luapula and Northwestern). In each province, we selected sites contributing 80% to provincial TB notifications: a mix of primary, secondary and tertiary level health facilities. Facilities were ranked in descending order of notifications and included sequentially until 80% of provincial TB notifications were captured. Among the selected facilities, none declined participation.

Routinely, household contacts of bacteriologically confirmed TB are symptomatically screened, and those with TB symptoms submit samples for further investigations for TB. Those diagnosed with TB are put on treatment while eligible contacts are initiated on TPT.

### Surge intervention procedures

This intervention was guided by elements of the Exploration, Preparation, Implementation, and Sustainment (EPIS) framework [10,11], which structures intervention planning across sequential phases and contextual layers. In the exploration stage, we used a needs assessment approach to identify gaps and contextual factors affecting TPT uptake among HIV negative contacts aged ≥ 5 years old. In the preparation stage, we developed strategies to leverage facilitators and address barriers, ensuring readiness for implementation. During the implementation stage, we delivered activities with attention to fidelity and adaptability. For the sustainment stage, we planned to support integration of effective interventions into routine practice after the surge. In this paper, we focus on the pre-surge (exploration and planning) and intra-surge (implementation) phases of the surge, highlighting their role in guiding planning and operationalization within the target context.

#### Pre-implementation.

We developed a TPT surge concept note in consultation with the National Tuberculosis and Leprosy Program (NTLP) and set targets. The targets were set a priori with the assumption that refusal rates would be higher among HIV negative contacts aged ≥5 years old. We targeted TPT initiation for at least 2,000 HIV negative household contacts to bacteriologically confirmed TB patients aged ≥5 years.

We conducted community engagement activities including media campaigns, door-to-door sensitization, and community meetings. We engaged MoH staff, community-based volunteers, and local gatekeepers to promote TPT uptake and address myths and misconceptions in the community. We also assessed and mobilized TPT commodities to ensure a steady supply across facilities during the surge.

The project team also oriented health care workers and community-based volunteers (CBVs) in TPT guidelines and surge targets and implementation approaches to ensure a uniform delivery. Additionally, copies of TPT guidelines and job aids were distributed to all participating sites as reference documents. To facilitate surge monitoring and easy abstraction of data from registers, we generated and piloted a DHIS2 tracker-based digital reporting tool.

#### Implementation procedures.

While implementation capacity may have varied across provinces and facility levels, the intervention package was standardized and delivered through existing National TB Programme structures, with technical support tailored to local needs. Two groups of household contacts of bacteriologically confirmed index TB cases were targeted: 1) contacts of TB patients diagnosed within three months prior to the surge 2) contacts of newly diagnosed TB patients during the surge. The surge primarily utilized a community-based approach, with multidisciplinary teams (each team comprising a nurse/clinician, a CBV and a Community Mobilization Officer where necessary), coordinated by local health facility staff, conducting home visits for contact investigation and community TPT initiation. We also provided TPT to walk-in clients at health facilities.

Symptom-based screening followed by GeneXpert testing for those with symptoms consistent with the definition of presumptive TB to rule out active disease, in line with recommendations for high TB burden and resource limited settings [5,12]. TB infection tests were not conducted, as these are not routinely available or offered during TB contact investigation in Zambia. Similarly, chest radiography is not universally accessible and was, therefore, not used as a mandatory test before offering TPT as guided by the WHO [13]. Contacts presenting with TB-related symptoms were classified as presumptive TB cases, and sputum or stool specimens were collected within the community for bacteriological testing at designated health facilities. Where health conditions required facility level evaluation, contacts were referred to health facilities for further clinical evaluation, including bacteriological confirmation and/or chest radiography where available. Presumptive TB contacts diagnosed with active TB were initiated on TB treatment, while those in whom TB was ruled out were subsequently reassessed for TPT eligibility and reintegrated into the care cascade.

Eligible contacts were counseled on possible TPT options according to the Zambian guidelines. The counseling provided information on TPT and the range of possible regimens, including duration, dosing frequency, and potential adverse effects. Contacts were then invited to select their preferred regimen. Where a preferred regimen was not available, health care workers offered alternative available regimens, and contacts who accepted were initiated on the alternative regimen. Contacts whose preferred regimen was unavailable were put on a waiting list until the preferred options were secured by the health facility.

We sustained demand generation activities during the surge through radio programmes and community engagement meetings addressing emerging reasons for TPT refusals.

We conducted daily monitoring and supervision to ensure surge implementation with fidelity and tracking of key indicators (TPT eligibility, initiation rates, refusal rates, and commodity stock levels). Furthermore, we held weekly progress review meetings and shared reports with key stakeholders: the MoH and USAID. We documented all activities, using a journal, to track implementation components potentially key to effective TPT rollout to the HIV negative at-risk populations ≥5 years old in routine practice.

### Data collection methods

The TPT surge data collection spanned the TPT surge implementation period from 1^st^ October 2024 to 15^th^ January 2025. Facility based MoH staff routinely documented surge data in facility-based registers while project data associates collected and entered the aggregate data into the electronic DHIS2 based tool weekly. Additionally, we extracted routinely collected programme data from the Facility Information Management System (FIMS) from 1^st^ January to 30^th^ September 2024 (pre-intervention period) and 16^th^ January to 30^th^ September 2025 (post intervention period) to inform analysis of the impact of the surge on TPT initiation trends.

### Data management and analysis

We extracted aggregate TPT surge data from the electronic data collection tool into excel and conducted descriptive analysis. The team analyzed intra-surge TPT initiation stratified by age category, sex as well as TPT regimen and presented it in the form of counts and proportions. Additionally, a segmented interrupted time series analysis was conducted using monthly program data from January 2024 to September 2025 to evaluate the association between the surge and TPT coverage among ≥5 years old HIV-negative household contacts of bacteriologically confirmed TB index cases. The analysis included three periods: pre-intervention (January–September 2024), intervention implementation (October 2024–Mid-January 2025), and post-intervention (January–September 2025). Autocorrelation was assessed using the Durbin Watson statistic, with values approximating 1.29 considered as no suggestion of first order autocorrelation. The interrupted time series model was estimated using the Newey West standard errors with a lag specification of 0 [lag (0)] to account for any serial correlation Segmented regression models estimated baseline level and trend, immediate level changes at the beginning of the surge and at the transition to post-intervention, and trend changes during intervention and post-intervention phases. Furthermore, information from decline forms was extracted to understand the common reasons for refusal of TPT.

### Definition of terms in the context of this study

i. **Presumptive TB patients** – A contact presenting with any one of the following symptoms: cough, fever, night sweats, shortness of breath, fatigue and chest pains. In children, symptoms also includes anorexia, poor weight gain/weight loss and reduced activity or playfulness [12].
ii. **A household contact** – a person who has shared an enclosed space for one or more nights or an extended period of time during the day with a person who has active TB in the past 3 months (MoH TPT guidelines).
iii. **TPT Eligibility** was based on the following [3]:
  - Contacts who screened negative for TB using the recommended WHO four symptom-based TB screening
  - Symptomatic contacts in whom TB was ruled out using GeneXpert tests
  - Contacts in whom active TB was ruled out and had no contra-indications
iv. **TPT Refusal** – Contacts eligible for TPT who declined treatment initiation for reasons other than availability of preferred regimen after at least two engagements during the surge.
v. **TPT Acceptance** - Contacts eligible for TPT expressing willingness to commence treatment using their preferred regimen.
vi. **TPT Initiation** – Eligible contacts who accepted to take TPT and commenced treatment during the surge

### Ethics statement

Ethics approval for the study was obtained from University of Zambia Biomedical Research and Ethics Committee, Lusaka, Zambia (Reference number 1773–2021) to analyze existing programme data. The University of Zambia Biomedical Research and Ethics Committee, Lusaka, Zambia (Reference number 1773–2021) also waived the requirement of written informed consent, as the analysis was retrospectively performed using routine data which was de-identified before analysis.

## Results

**Fig 1:**
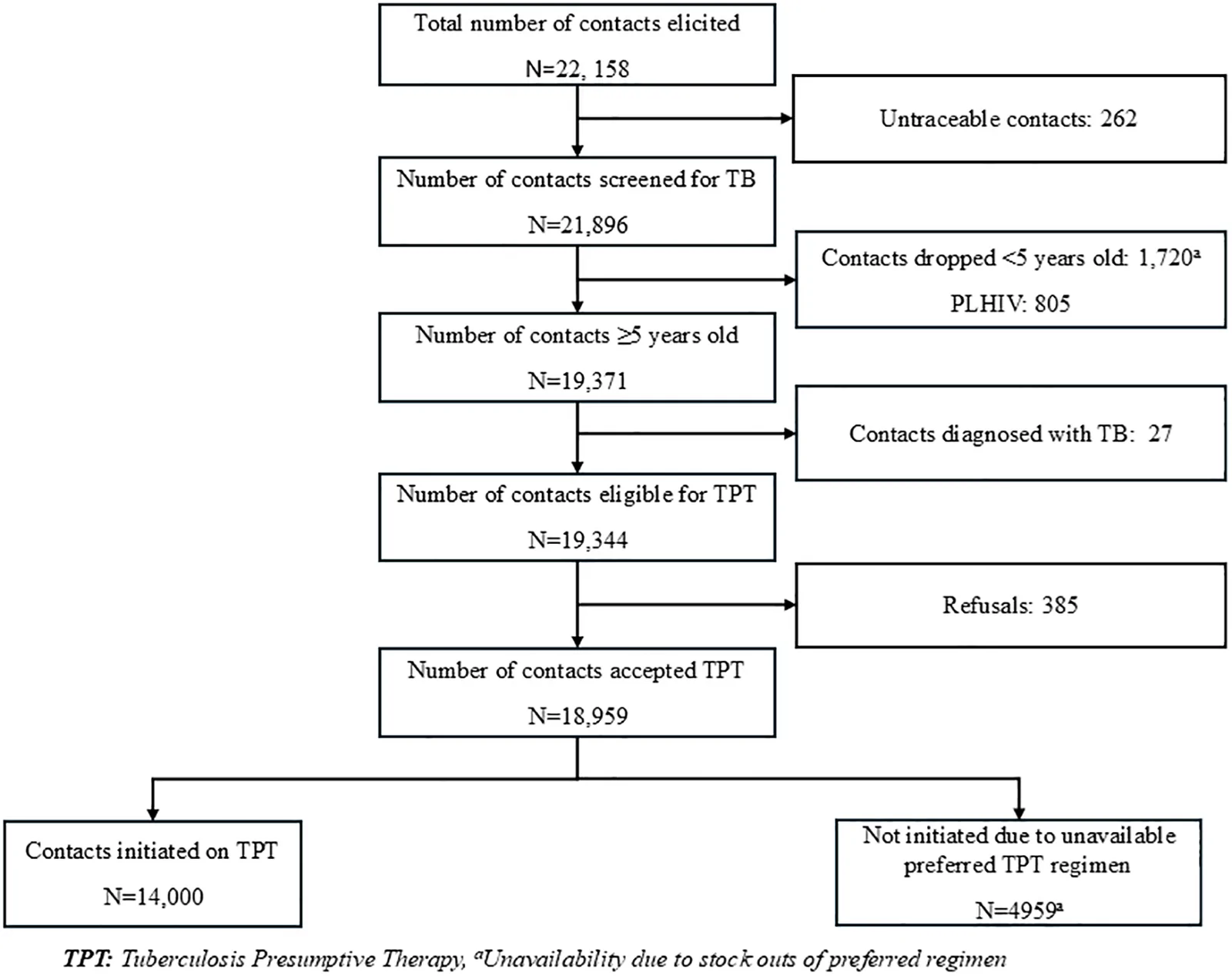
TPT surge flow chart for TB contacts.

Overall, 22,158 household contacts were enlisted during the surge among whom 98.8% (n = 21,896) were traceable (Fig 1). Of the traced contacts, 88.5% (n = 19, 371) were HIV negative aged ≥5 years. Of these, 99.9% (n = 19,344) were eligible for TPT among whom 2.0% (n = 385) declined TPT. Among 18,959 contacts who accepted TPT, 26.2% (n = 4,959) were not initiated on treatment by the end of the surge due to unavailability of their preferred regimen. A total of 14,000 ≥ 5 years old HIV negative contacts (72.4% of those eligible and 73.8% of those who accepted TPT) were commenced on treatment.

Of the contacts screened (Table 1), we identified 6.8% (n = 1,316) presumptive TB cases among whom 2.0% (n = 27) were diagnosed with TB using GeneXpert tests and initiated on treatment.

**Table 1:** Age disaggregated TPT cascade for HIV negative contacts to Bacteriologically confirmed TB aged ≥ 5 years (n (%)) screened for TB.

Age category	Contacts Screened	Presumptive TB cases identified	Number Diagnosed with TB	Eligible For TPT	Initiated On TPT
5-14	4846 (97.6)	302 (6.2)	4 (1.3)	4842	3414 (70.5)
15-64	13927 (99.3)	959 (6.9)	21 (2.2)	13906	10139 (72.9)
65+	598 (96.5)	55 (9.2)	2 (3.6)	596	447 (75.0)
Total	**19,371 (98.8)**	**1,316 (6.8)**	**27 (2.0)**	**19, 344**	**14000 (72.4)**

Percentages are row percentages.

Among the 14,000 HIV negative contacts aged ≥ 5 years old who were initiated on TPT (Table 2), majority (72.4%, n = 10139) were aged 15–64 years and 52.7% (n = 7378) were female. The majority received TPT at level one hospitals (40.8%, n = 5718) and urban health centres (30.6%, n = 4280).

**Table 2:** Characteristics of HIV negative Contacts initiated on TPT (N = 14000).

Variable	Number of Contacts *n(%)*
Age group (years)	
5-14	3414 (24.4)
15-64	10139 (72.4)
≥ 65	447 (3.2)
Sex, *n (%)*	
Male	6622 (47.3)
Female	7378 (52.7)
TPT initiating facility type, *n (%)*	
Third Level	860(6.1)
Second Level Hospital	2005 (14.3)
First Level Hospital	5718(40.8)
Urban HC	4280(30.6)
RHC	1137(8.1)

Overall, most contacts (63.7%, n = 8917) were initiated on the three months Isoniazid and Rifapentine based regimen (3HP), followed by six months Isoniazid (6H) (30.2%, n = 4,226); one month Isoniazid and Rifapentine (1 HP) (4.5%, n = 625,) and the least on three months Isoniazid and Rifampicin (3HR) (1.7%, n = 232) (Table 3).

**Table 3:** Types of TPT regimens initiated for eligible TB contacts, disaggregated by age, n(%).

Age category	*N*	6H	1HP	3HP	3HR
5-14	3,414	1379 (40.4)	0 (0.0)	1963 (57.5)	72 (2.1)
15-64	10,139	2756 (27.2)	609 (6.0)	6639 (65.5)	135 (1.3)
65+	447	91 (20.4)	16 (3.3)	315 (70.5)	25 (5.6)
Total	**14,000**	**4226 (30.2)**	**625 (4.5)**	**8917 (63.7)**	**232 (1.7)**

Percentages are row percentages with N as denominator; 6H – 6 months Isoniazid; 1 HP – 1 month Isoniazid + Rifapentine; 3HP – 3 months Isoniazid + Rifapentine; 3HR – 3 months Isoniazid + Rifampicin.

**Fig 2:**
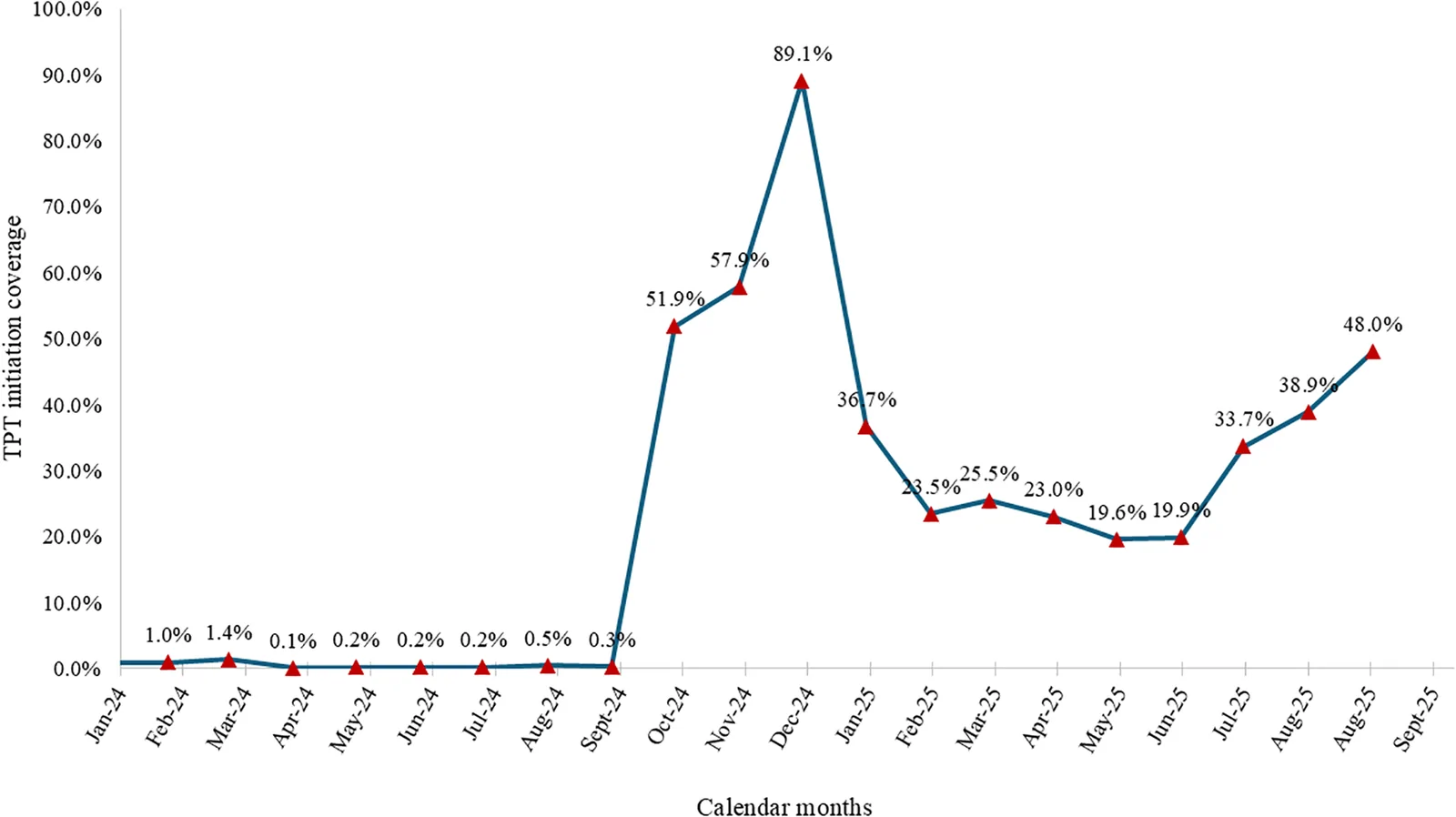
Pre- intra- and post implementation TPT Coverage trends in TB LON supported sites (January 2024 to September 2025).

Compared with pre-surge programme data, TPT coverage among HIV negative contacts aged ≥5 years ranged from 0% to 1.4% between January and September 2024 (Unpublished routine data) (Fig 2). During the surge period (1^st^ October 2024–15^th^ January 2025), TPT coverage increased sharply from a baseline of 0.3% in September to 51.9% in October, 57.9% in November, 89.1% in December before declining to 36.7% in January 2025 and ranging from 23.5% to 48.0% between February and September 2025.

After the surge, TPT coverage among contacts aged ≥5 years declined but did not return to the pre-intervention status and showed an upward trend after June 2025.

**Fig 3:**
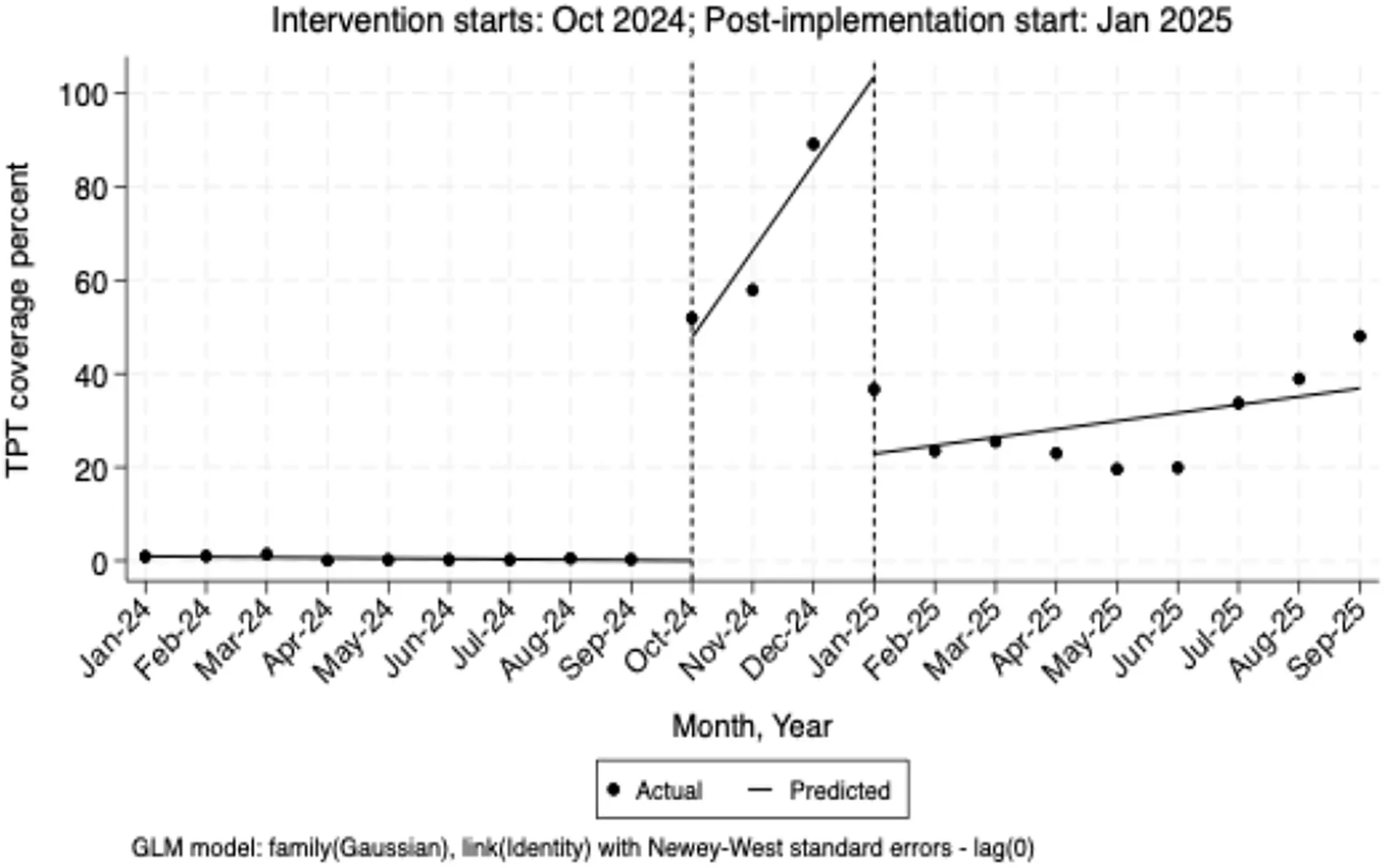
Interrupted Time Series of TPT coverage showing the start of the scale-up intervention in October 2024 and the beginning of the post-implementation period in January 2025.

For the interrupted time series analysis (Fig 3), the assessment of autocorrelation using the Durbin Watson yielded a value of 1.29, suggesting absence of evidence of first order autocorrelation. Therefore, the interrupted time series analysis used Newey West standard errors with lag specification of 0 because no serial correlation was detected.

The interrupted time series analysis (Table 4) showed that before the start of the TPT surge, coverage was low and declined slightly over time by 0.10% points per month (p = 0.006). At the start of the intervention, there was an immediate increase in coverage of approximately 47.7% points (p = 0.001). Coverage continued to rise thereafter, increasing by about 18.7% per month (p = 0.001) relative to the pre-intervention trend. In the post intervention period, there was a marked immediate decline of approximately 80.6% points (p = 0.001) and a monthly decline of 16.9% points per month (p = 0.001), relative to the intervention period. Based on the coefficients, the post intervention slope was + 1.6.

**Table 4:** Intervention Interrupted Time Series analysis of the impact of the surge on TPT coverage trends.

Variable	Coef.	Newey-West Std. Err.	t	p > |t|	[95% Conf. Interval]
Baseline pre-intervention slope	−0.10	0.04	−2.76	0.006	−0.18–0.03
Immediate level change in coverage at intervention start	47.7	5.37	8.88	0.001	37.16–58.21
Change in slope during the surge	18.6	3.51	5.32	0.001	11.82–25.59
Immediate level change at post-intervention start	−80.6	10.58	−7.68	0.001	−101.35–59.88
Change in slope during the post intervention relative to intervention period	−16.9	3.80	−4.44	0.001	−24.30–9.41

Among contacts who declined TPT (Table 5), the most frequently reported reason was concerns about side effects (30.3%, n = 136). Fear of stigma or discrimination was the second most common reason (17.1%, n = 77), followed by contacts taking other medications (14.7%, n = 66) and a perceived low personal risk of TB (11.1%, n = 50).

**Table 5:** Reported reasons for declining TPT among eligible contacts.

Reasons For Declining TPT	Count of Reasons For Declining TPT	Frequency (%)
Concerns about side effects	136	30.3%
Fear of stigma or discrimination	77	17.1%
Currently taking other medications	66	14.7%
Perceived low personal TB risk	50	11.1%
Lack of understanding of TPT benefits	37	8.2%
Lack of trust in healthcare system	15	3.3%
Family refused	14	3.1%
Religious beliefs	9	2.0%
Previous negative experience with TB treatment	5	1.1%
Cost related concerns	1	0.2%
Cultural beliefs	1	0.2%
Missing data	38	8.4%

Limited understanding of the benefits of TPT accounted for 8.2% (37) refusals (S2 Table). Less commonly cited reasons included lack of trust in the healthcare system (3.3%, n = 15), family refusal (3.1%, n = 14), religious beliefs (2.0%, n = 9), previous negative experience with TB treatment (1.1%, n = 5), cost-related concerns and cultural beliefs (1 each; 0.2%). Reasons for declining TPT were missing for 38 participants (8.4%).

## Discussion

This analysis revealed that by the end of the surge implementation, there was a statistically significant increase in TPT initiation among HIV-negative household contacts aged ≥5 years from a baseline of 0.3% before the surge to 89.1% within three months, exceeding historical national average and surpassing the WHO-recommended 85% coverage threshold among household contacts [14,15]. This single surge contributed over 70% to Zambia’s TPT initiation among household contacts in 2024 [16]. After the surge, a decline in TPT coverage was observed; albeit not returning to the baseline performance. This was the first implementation analysis in Zambia and regionally to demonstrate feasibility and potential effectiveness of a surge approach in scaling up TPT coverage among HIV-negative household contacts to bacteriologically confirmed TB aged ≥5 years.

This intervention demonstrated that implementing a well-planned, well-designed and executed TPT surge targeting all TB household contacts was feasible and is associated with a statistically significant increased TPT uptake among HIV negative contacts aged ≥5 years: a population historically under-prioritized in TB prevention programming. The success of the TPT surge can be attributed to adequate planning, healthcare workers capacity building, close performance monitoring as well as community engagement and demand generation pre- and intra-surge. Activities such as health education potentially contributed to increased uptake by addressing low risk perceptions, fear of side effects, beliefs, myths and misconceptions and other documented barriers to TPT acceptance, especially among adults [17]. Furthermore, integrating trained community-based volunteers and local champions into community engagement and service delivery may have improved trust and supported TPT acceptance. This aligns with growing evidence that community-based delivery models coupled with systematic contact tracing and decentralized service delivery, can be associated with improved access and uptake of TB services in high-burden settings [18–20] especially if delivered by adequately capacity built multi-disciplinary teams, as was done in this surge, and adequately resourced [4].

While TPT provision in Zambia, like other low income countries, has remained skewed toward PLHIV and ≤5 years old children [21], the HIV-negative contacts ≥5 years old have remained largely underserved despite constituting the majority of household contacts [7]. As a result, the TB burden among HIV-negative individuals has largely remained static or has declined at a much slower rate over the past decades compared to the PLHIV [2]. Despite the overall TB risk remaining high among PLHIV [2,16], there is a decline in TB incidence in this group; a trend mainly attributable to the combined effect of antiretroviral therapy (ART) and TPT [1,22]. Considering the documented effectiveness of TPT in reducing incident TB [4,5], improving coverage can help reduce TB morbidity and community spread among this population; critical ingredients for ending TB [4,7,23]. Failure to prevent active TB in HIV-negative individuals can have substantial long-term consequences. Strong immune responses during disease often lead to greater structural lung damage, including cavitation, fibrosis, and bronchiectasis, resulting in post-TB lung disease (PTLD). Approximately 20%–45% of TB survivors develop PTLD, with prevalence generally higher among HIV-negative individuals than among PLHIV [24–26].

Our findings challenge the assumption that low TPT uptake in this group is due to poor acceptability [27]; instead suggesting that previous low coverage may be a function of system-level weaknesses such as ineffective TPT delivery approaches and a lack of prioritization rather than low acceptability among targets. This aligns with previous qualitative studies among health care workers in South Africa that established that HCWs’ negative attitudes and system weaknesses such as poor contact investigation strategies and resource shortages are key drivers of low TPT uptake among TB contacts [17]. Therefore, more investment and system strengthening are required to improve TPT uptake among all TB contacts regardless of age and HIV status.

Despite TPT initiation rate being constrained by system weaknesses, mainly TPT medicines availability, with about 26.2% of the contacts accepting TPT but failing to commence treatment due to unavailability of their preferred regimen, the high initiation rate achieved during the surge (72.4%) demonstrates that substantially high acceptance rates are attainable when implementation barriers such as low-risk perception [17] are effectively addressed. This suggests that pre-surge concerns about potential poor uptake were overstated. Notably, the refusal rate observed among HIV-negative household contacts aged ≥5 years was only 2.0%, highlighting the potential acceptability of TPT in this demographic when interventions are well planned, executed with fidelity, community-focused and adequately resourced. This contrasts with earlier studies that reported markedly lower acceptability among HIV-negative household contacts aged ≥5 years, in some cases as low as 3% [1,28]. However, similar to those earlier studies in South Africa and Uganda, fear of side effects, misconceptions, community stigma, and low risk perception were cited as key barriers in this intervention [17,29,30]. This highlights the need for tailored counselling that clearly communicates the risk associated with contact with TB, the importance of TPT, expected adverse events, their frequency, and management, alongside proactive early follow-up. Overall, expanded TPT access must be paired with targeted health education, stigma reduction, and integrated care models to address both perceived and structural barriers.

This analysis suggests that rolling out TPT to HIV-negative contacts aged ≥5 years is feasible. The surge leveraged existing national TB program infrastructure, clinics, laboratories, equipment, commodities, drugs, and human resources, to deliver services, requiring only additional support for site orientations, data management, transport logistics and technical support supervision from the project. The rapid increase in TPT initiation from 0.3% to 89.1% shows that high coverage among HIV-negative household contacts aged ≥5 years is operationally achievable in Zambia when services are well planned, resourced, and closely monitored with strong community engagement. The observed decline in TPT coverage immediately after the surge highlights a sustainability challenge rather than poor acceptability.

The observed reduction in TPT initiation post surge is mostly attributable to the impact of the United States Government stop work order which saw a withdrawal of logistical and Technical support for TB activities. This happened immediately after the end of the surge; with no room for effecting routinization of activities. A sudden withdrawal of technical and financial support before routinizing the intervention caused a shock in the delivery of TB services within the community using the multi-disciplinary teams resulting in a decline in TPT initiation. Additionally, the project supported close monitoring of performance slowed down. This reinforces the need for a phased transitioning of donor support to the Ministries of Health to prevent operational shocks that impact on performance [31,32]. The observed rise in TPT initiation between June and September 2025 coincided with the project resumption of TB LON activity implementation in the supported provinces after the stop work order emphasizing the magnitude of partner support in the delivery of TPT.

Although commodity shortages during the surge may have influenced regimen availability, the distribution of TPT regimens revealed a pronounced preference for the short-course rifapentine-based 3HP regimen (63.7%), particularly among adults. The preference for 3HP over 1 HP and other available regimen was mainly due to the convenience of administration - once weekly compared to daily administration of the other options [33]. Additionally, limited drug availability during the implementation period may have contributed to a lesser extent. This preference is supported by WHO’s 2020 recommendations, which grant 3HP strong endorsement due to its non-inferior efficacy, convenience of administration, and lower hepatotoxicity compared to longer isoniazid regimens [3,20]. Moreover, nearly 25% of the TB contacts could not commence TPT in our analysis despite their willingness to take it due to unavailability of the preferred shorter regimen. Studies have shown higher completion and adherence rates for 3HP compared to longer regimens like 6H [34–36]. Recent systematic reviews of completion rates of 3HP relative to other treatment regimens for latent tuberculosis infection revealed that shorter, 3–4 months long, regimens were more likely to be completed than longer regimens [37,38]. Real-world implementation in Uganda among PLHIV confirmed these findings: acceptance and completion exceeded 90% across different delivery strategies [39]. Collectively, these findings suggest that when supply permits, patients and providers strongly favor the shorter, more patient-friendly 3HP regimen. Service providers must therefore ensure reliable procurement and supply-chain resilience to maintain access to rifapentine-based TPT regimen.

Even though Zambia’s national TB incidence remains high overall (283/100,000) [1], the notable recent decline in TB among PLHIV mainly attributed to ART and TPT scale-up in this population [1,22,40] can be replicated in the HIV negative population. Scaling up TPT nationally among HIV negative contacts to bacteriologically confirmed TB using the strategies tested in this surge could help flatten the TB incidence curve among this population by reducing community transmission, as previously modelled in other high-burden settings [5,22,41]. Moreover, this initiative also presents a replicable model for other high-burden countries aiming to operationalize the WHO’s person-centered and community-oriented TB prevention guidance [3]. The improved TPT coverage among HIV negative contacts in this surge activity aligns with global TB elimination goals outlined in the UN High-Level Meeting Declaration [42], which calls for inclusive, equitable, and context-appropriate delivery of preventive services to all at-risk populations, including HIV-negative individuals.

Despite the successes of this study, it did not lack limitations. Firstly, only symptom screening and GeneXpert tests were used to rule out TB with a possibility of missing asymptomatic TB. However, as recommended by WHO, the absence of TB infection tests and chest X-rays did not hinder TPT initiation [12]. Secondly, this analysis relied on routinely collected aggregate data, which may be subject to reporting inaccuracies, over-reporting or underreporting and limit the analyses that could be conducted. Thirdly, the interrupted time series analysis was based on 21 monthly observations with only four observations during the intervention period. The limited number of time points within this segment may affect the precision of the estimates within segment trends and statistical power to detect changes over time. Therefore, the intervention trend estimates should be interpreted cautiously because they are based on few observations, resulting in wide uncertainty around the slope estimates and limiting confidence in whether the observed trends represent true intervention effects or random temporal variation. Fourthly, TPT completion outcomes were not systematically collected and included in this analysis. Consequently, our findings should be interpreted as reflecting programme success in TPT initiation rather than the full effectiveness of TPT delivery, which requires both initiation and completion to achieve optimal protection against progression to active TB disease. Nonetheless, the statistically significant increase in uptake following implementation provides strong suggestive before and after evidence of the intervention’s effectiveness and a proof of concept for scaling up TPT to HIV negative contacts ≥5 years old in Zambia and elsewhere. Further, limited sustainment following cessation of surge support as a key limitation, particularly in the context of the sudden withdrawal of all resources including funding and human resources before routinization could be achieved.

## Conclusion

The TPT Surge demonstrated that with intentional design, staff capacity building, strong community engagement, and real-time data monitoring, it is feasible to achieve high TPT coverage among HIV-negative contacts aged ≥5 years in Zambia and beyond. These results support national and global calls for inclusive, person-centered TB prevention strategies that do not exclude older HIV-negative individuals. As Zambia moves to institutionalize its TPT roadmap, integrating surge elements into routine programming could accelerate progress toward TB elimination.

## Supporting information

S1 Data TPT surge data. (XLSX)

S1 Table TPT trends. Pre- intra- and post implementation TPT Coverage trends for eligible contacts ≥5 years old in TB LON supported sites (January 2024 to September 2025).
(XLSX)

S2 Table TPT refusal reasons. Reasons given by eligible contacts for declining TPT during the surge.
(XLSX)

S3 Table Reported data definitions. Definitions of denominators and numerators for the key variables reported.
(XLSX)
